# Exploring Perceived Barriers and Facilitators of PrEP Uptake among Young People in Uganda, Zimbabwe, and South Africa

**DOI:** 10.1007/s10508-020-01880-y

**Published:** 2021-05-05

**Authors:** Richard Muhumuza, Andrew Sentoogo Ssemata, Ayoub Kakande, Nadia Ahmed, Millicent Atujuna, Mangxilana Nomvuyo, Linda-Gail Bekker, Janan Janine Dietrich, Gugulethu Tshabalala, Stefanie Hornschuh, Mamakiri Maluadzi, Lynda Chibanda-Stranix, Teacler Nematadzira, Helen Anne Weiss, Stephen Nash, Julie Fox, Janet Seeley

**Affiliations:** 1 Medical Research Council/Uganda Virus Research Institute and London School of Hygiene & Tropical Medicine Uganda Research Unit, P.O. Box 49, Entebbe, Uganda; 2 Desmond Tutu HIV Foundation, Faculty of Health Sciences, University of Cape Town, Cape Town, South Africa; 3 Perinatal HIV Research Unit, School of Clinical Medicine, Faculty of Health Sciences, University of the Witwatersrand, Johannesburg, South Africa; 4 Health Systems Research Unit, South African Medical Research Council, Bellville, Cape Town, South Africa; 5 Clinical Trials Research Centre, University of Zimbabwe, Harare, Zimbabwe; 6 MRC International Statistics & Epidemiology Group, Department of Infectious Disease Epidemiology, London School of Hygiene & Tropical Medicine, London, UK; 7 Department of Infectious Diseases, King’s College London, London, UK; 8 Department of Global Health and Development, London School of Hygiene & Tropical Medicine, London, UK

**Keywords:** PrEP, Adolescents, Barriers, Facilitators, HIV prevention

## Abstract

Pre-exposure prophylaxis (PrEP) is an effective HIV prevention strategy. Few studies have explored adolescents and young people’s perspectives toward PrEP. We conducted 24 group discussions and 60 in-depth interviews with males and females aged 13–24 years in Uganda, Zimbabwe, and South Africa between September 2018 and February 2019. We used the framework approach to generate themes and key concepts for analysis following the social ecological model. Young people expressed a willingness to use PrEP and identified potential barriers and facilitators of PrEP uptake. Barriers included factors at individual (fear of HIV, fear of side effects, and PrEP characteristics), interpersonal (parental influence, absence of a sexual partner), community (peer influence, social stigma), institutional (long waiting times at clinics, attitudes of health workers), and structural (cost of PrEP and mode of administration, accessibility concerns) levels. Facilitators included factors at individual (high HIV risk perception and preventing HIV/desire to remain HIV negative), interpersonal (peer influence, social support and care for PrEP uptake), community (adequate PrEP information and sensitization, evidence of PrEP efficacy and safety), institutional (convenient and responsive services, provision of appropriate and sufficiently resourced services), and structural (access and availability of PrEP, cost of PrEP) levels. The findings indicated that PrEP is an acceptable HIV prevention method. PrEP uptake is linked to personal and environmental factors that need to be considered for successful PrEP roll-out. Multi-level interventions needed to promote PrEP uptake should consider the social and structural drivers and focus on ways that can inspire PrEP uptake and limit the barriers.

**Electronic supplementary material:**

The online version of this article (10.1007/s10508-020-01880-y) contains supplementary material, which is available to authorized users.

## Introduction

Pre-exposure prophylaxis (PrEP) is a promising and effective HIV prevention strategy, utilizing antiretroviral (ARV) medication taken as a single oral pill to prevent the acquisition of HIV (Golub, Gamarel, & Surace, 2017). The World Health Organization (WHO, 2012) approved PrEP as an additional biomedical prevention strategy and released guidelines for PrEP use among particular groups of people, i.e., sero-discordant couples and men who have sex with men (MSM). More recently, the WHO has broadened the recommendation to include all groups at substantial risk of HIV infection (World Health Organization, 2015).

Adolescents and young people (AYP) aged 15–24 years account for approximately 30% of all new HIV infections worldwide with adolescent HIV most prevalent in Africa (Joint United Nations Programme on HIV/AIDS, 2015; Rotheram-Borus, Davis, & Rezai, 2018). This could be attributed to the multiple co-occurring transitions during adolescence such as increased autonomy, decreased adult supervision, identity formation, peer influence, and social transition, potentially leading to early sexual debut and health risk behaviors that may lead to HIV infection (Cicchetti & Rogosch, 2002; Schulenberg, Maggs, & Hurrelmann, 1999). Despite this, the levels of PrEP acceptability and uptake among AYP in sub-Saharan Africa remain unclear, as research on PrEP has focused on high-risk adult populations (Bärnighausen et al., 2019; Shah, Gillespie, Holt, Morris, & Camacho-Gonzalez, 2019; Vaughn, Dillon, & Kedia, 2019; World Health Organization, 2017b). Previous work among MSM showed that stigma, drug effects, and adherence were among the barriers to PrEP uptake (Holloway et al., 2017; Owens et al., 2020), while a facilitator of uptake was the provision of PrEP free of charge (Golub et al., 2017). These barriers and facilitators did not differ in the population of women and populations most at risk in East Africa and other countries (Amico et al., 2017; Mack, Odhiambo, Wong, & Agot, 2014; Thigpen et al., 2012; Young, Flowers, & McDaid, 2014).

As part of the strategies to attain the 95-95-95 fast-track action to end the AIDS epidemic by 2030, engaging AYP in the co-creation of HIV prevention strategies, including PrEP, needs to be rapidly adopted in sub-Saharan settings such as Uganda, Zimbabwe, and South Africa with growing AYP populations to successfully reduce the epidemic (United Nations, 2012; United Nations General Assembly, 2016; World Health Organization, 2017a). This is critical as many of these countries have large adolescent populations at high risk of HIV infection with high HIV incidence rates (Baeten et al., 2012; Shisana et al., 2014; World Health Organization, 2012). In this study, we sought to explore the barriers and facilitators of uptake of PrEP among AYP, to help formulate and inform PrEP implementation activities for this age group. This will support the development of cultural-, age-, and gender-appropriate risk reduction understanding for adolescents and young people 13–24 years at risk of HIV acquisition.

### Theoretical Orientation

A growing body of literature shows that health outcomes are increasingly influenced by the environments within which individuals live and less by individual behavior (Busza et al., 2012; Feldacker, Ennett, & Speizer, 2011; Krieger, 2001). We therefore adopted the social ecological model (SEM) as a theoretical framework for analysis. The SEM has five interdependent levels that offer a holistic approach to understanding how individual, interpersonal, community, organizational, and structural factors influence health behaviors (McLeroy, Bibeau, Steckler, & Glanz, 1988; Stokols, 1996), including those associated with HIV prevention (Batchelder, Gonzalez, Palma, Schoenbaum, & Lounsbury, 2015; Joore, van Roosmalen, van Bergen, & van Dijk, 2017; Kaufman, Cornish, Zimmerman, & Johnson, 2014). The first level refers to individual factors that facilitate or inhibit a person’s choices, including personal stigma, fear of HIV, and personal adherence challenges. The second level is interpersonal/network influences. An individual’s relationship with their closest social peers, partners, and family members influences the AYP’s uptake of PrEP. The third level is community perspectives, as young people are influenced by community-held fears of PrEP side effects, and myths and misconceptions. The fourth level refers to health system (institutional) influences, including busy, unapproachable health workers and long waiting times at clinics. The final level refers to structural influences including the accessibility of PrEP, and long distances to clinics and school. AYP operate within these structures, and their choices are based on their motivations and interests (Barnett, Seeley, Levin, & Katongole, 2015; Kaufman et al., 2014).

Drawing upon the SEM framework, we offer theoretically informed insights regarding the potential barriers and facilitators associated with providing this form of prevention to PrEP-naive AYP populations.

## Method

### Participants

This study was part of the Combined HIV Adolescent PrEP and Prevention (CHAPS) study evaluating the acceptability and feasibility of providing daily and on-demand PrEP to adolescent boys and girls in the three countries (South Africa, Uganda, and Zimbabwe) conducted between September 2018 and February 2019. We conducted 24 group discussions (GDs) with 6–8 participants (8 per country; 4 for each gender) and 60 in-depth interviews (IDIs) (20 per country) stratified by age (13–17 and 18–24 years) and gender (males and females) to enhance the possibility of open discussion among young people of the same age and gender.

Krueger (2014) recommends GDs consisting of 6–8 participants when conducting discussions on topics that are non-sensitive in order to encourage information generation through group participants interaction. As a method of data collection, GDs encourage open, interactive discussion of shared norms, beliefs and common knowledge. However, GDs inherently facilitate in-depth discussion of individual standpoints within the context of other group members and may over represent opinions of participant(s) dominating the discussion (Krueger, 2014). We therefore conducted IDIs in order to increase reliability of our data.

One-to-one IDIs with a small number of AYP were conducted to obtain thick in depth descriptions and valuable insights about the barriers and facilitators to PrEP uptake. These interviews were conducted to provide further context, perspectives and more detailed information alongside the FGD data. IDIs were helpful to obtain information from AYP who may be uncomfortable sharing their honest feelings in the group setting (Ritchie, Lewis, Nicholls, & Ormston, 2013).

GDs and IDIs were conducted by a gender matched moderator to guide the data collection process as a formative evaluation means to explore AYP’s barriers and facilitators of PrEP uptake. Participants were recruited through purposive community sampling with the help of community mobilisers. The IDIs and GDs were held in a conducive place that was safe, neutral and with minimal distractions for the participants and the researchers. The demographic composition of the study participants is presented in Table 1.

**Table 1:** Demographic characteristics of study participants

Variable	In-depth interviews		Group discussions	
	Females (n = 31)	Males (n = 29)	Females (n = 84)	Males (n = 83)
Age in years				
Mean (SD)	18.23 (3.262)	17.58 (3.166)	18.26 (3.732)	18.64 (3.604)
Education				
Primary and below	08	08	21	12
Secondary	14	15	45	48
Tertiary	03	03	05	13
Preferred not to share	06	02	13	10

At the time of the study in 2018/2019, Uganda had not rolled out PrEP to the general population, while in Zimbabwe, PrEP for AYP was only available at selected sites with one such site within the study recruitment area, and for South Africa PrEP was available to selected high-risk groups. Most participants were PrEP-naïve, and PrEP was not readily available to young people in the three countries. Most participants received information about PrEP for the first time during mobilization and implementation of this research.

### Procedure

Experienced social science researchers conducted the GDs and IDIs, using a semi-structured topic guide (Appendix A in electronic supplementary materials), in a secure and private location that was comfortable for the interviewer and the interviewee. This place was either suggested by the interviewee or preset by the interviewer at the local health clinic or community hall. The IDIs and GDs generally focused on motivations and hindrances for HIV testing, PrEP preference (daily vs on-demand), and barriers and facilitators of PrEP uptake. Data collection was conducted in a language comfortable to the participant, audio-recorded, transcribed verbatim and later translated into English.

Participants provided written informed consent for those above 18 years, and written assent and parental consent for those below 18 years. A waiver of parental consent was obtained for emancipated minors according to national guidelines from the regulatory authorities in the three countries. Participants were reimbursed for their time and participation in the study according to site-specific guidelines.

### Data Analysis

A framework approach using the SEM was used for data analysis (Gale, Heath, Cameron, Rashid, & Redwood, 2013). To achieve data familiarization, the researchers at each site read the transcripts several times and made notes of key ideas and recurrent codes. Four transcripts were initially coded to identify emerging and recurrent themes which were compared across sites to ensure consistency and refine the coding framework and codebook. It is at this point that all the remaining transcripts were coded using the refined codebook. Data were then indexed by identifying segments of the data that corresponded to a particular code.

The research teams discussed mapping and interpretation of the data to develop broader categories allowing them to distil the meaning of the data in context according to each research question. We present the overall analysis of data from the GDs and IDIs without country-specific analysis.

## Results

The majority of participants at the three sites were single, had attained secondary-level education, and those out of school were either running a commercial business, or casually employed (involved in menial jobs requiring minimal skills with no regular work pattern or, formal employment) and a few were unemployed. We found common barriers and facilitators to PrEP uptake at multiple levels from the three countries as presented below.

### Barriers to PrEP Uptake Among Adolescents and Young People

- Individual-level barriers

From the IDIs and GDs we found the following individual-level barriers to PrEP uptake: PrEP-related stigma, pill burden, doubting PrEP efficacy, the complexities of the timing, and schedule of taking PrEP and PrEP characteristics.

### Stigma Related to PrEP Uptake

The participants mentioned that they would refrain from taking PrEP because of the association of PrEP with antiretroviral drugs and HIV-related stigma. This was a key barrier to uptake as participants linked taking daily tablets to people living with HIV. Thus, taking medication, especially daily, can be mistaken for taking ARVs for treatment rather than for prevention. HIV-related stigma and stigma associated with being promiscuous were concerns raised.

There is a fear that people will think you are already HIV positive and you are taking ART because many people do not have enough information about PrEP. (IDI Male 22 years; Zimbabwe)

And [stigma] will make young people feel outcasts within the community because people will say that you are lying by saying that this thing [PrEP] is to prevent. We know you are already infected, that is what would discourage them...You know rumors spread faster than the truth...as soon as one person thinks that PrEP is for sick people then that’s what everyone will think. (GD Females 18–24 years, South Africa)

AYP suggested that they would take PrEP in secret so nobody would see them and where there is limited privacy, taking PrEP would become difficult. Participants also mentioned that they would not disclose their PrEP use to their peers, for fear of being talked about.

You have to hide. Sometimes you may go with your friends to the club and get a man. You may fear to take that PrEP because your friends are around or you may fear to take PrEP because your friends may think that you are promiscuous. (GD Females 20–24 years; Uganda)

### PrEP Pill Burden

Most of the participants expressed a fear of taking many tablets and thought that they would reach a time when they could no longer take the PrEP pills. It is at this point that participants gave scenarios to illustrate the circumstances that might make it hard for somebody to adhere to PrEP, such as already having other medications to take:

Some people have other illnesses. You may find one has to take medicines for pressure or diabetes. So if you add them PrEP, it may be a burden to her. (IDI Female 18 years; Uganda)

Pill fatigue created by taking PrEP tablets daily when you are not sick was a concern raised by participants as a barrier to PrEP uptake. This also has implications for PrEP adherence.

When you have HIV, you take ARVs daily. It feels the same any way you take PrEP every day. You take PrEP pills like you are sick but you are not sick…Some won’t want it [PrEP] because they are lazy to take pills, more so taking pills every day. (GD Males, 13–17 years; South Africa)

The AYP in our study associated taking medication with treatment or as a cure for a condition, rather than prevention. For some, taking medicine when healthy was thought to be strange. Other participants mentioned that they had a general dislike for swallowing pills, preferring injections, and this would be a barrier to adhering to oral PrEP:

Some can refuse PrEP because they do not like pills generally. Others don’t like to take pills on a daily basis especially when not ill and cannot cope with the pills so considering this some people can refuse them [PrEP pills]. (IDI Female 24 years; Zimbabwe)

### Doubting PrEP Effectiveness

Some participants raised doubts about the effectiveness of PrEP to prevent HIV infection and this was viewed as a potential barrier to uptake. Although there were participants who recognized the usefulness of PrEP as prophylaxis to prevent HIV if it worked, many were skeptical about its effectiveness:

Most HIV drugs are under investigation in clinical trials and most of them are found not to be effective when the trials are complete that is why I am still doubting PrEP effectiveness. Those of us who have received information about PrEP shall share it with others when we go back […] some can only accept PrEP after understanding its effectiveness but if not, they can’t accept it. (GD Males 18–24; Uganda)

What am I afraid of is that you see PrEP may not be working, it may not work effectively and may make the HIV situation worse, you see. (IDI Female 13 years; South Africa)

### Complexities of Timing of Taking PrEP

There was a perception that daily PrEP would require stringent time keeping and maintaining a regular routine. Some AYP explained that sexual activity was not necessarily planned with such regularity.

…at times you might be with your boyfriend and you left the pills at home and you want to have sex right there and then and you weren’t taking PrEP every day and it becomes difficult. (GD Females 19–21 years; Zimbabwe).

Participants also mentioned that some jobs are demanding and may not allow time to collect their medication or they may forget to take it. Some expressed concerns about unplanned work activities and the consequences if they missed their PrEP dose and engaged in sexual activity.

I think it won’t be easy for most people to use it [PrEP] because the nature of their jobs doesn’t give them time…A person may be having a lot of things to do and he/she forgets to take it yet you don’t know when you are going to have sex. (GD Males 13–17 years; Uganda)

### PrEP Characteristics

The concerns about PrEP tablets included the taste, smell, size, and possible side effects of the tablets.

### Odor and Taste of the Pill

Some participants expressed the view that the odor and taste of the pill may be a barrier to PrEP uptake. Participants said that a pill with no smell would be more acceptable.

I might swallow PrEP and it causes my tongue to taste like everything is sour or whatever I eat [afterwards] to taste sour…I do not want sour pills and that would stop me from taking PrEP. (GD Females 13–17 years; Uganda)

…when I take a pill every time, like if I take 1 pill for 5 days or 7 days, for me the taste changes. It starts having a bad taste, a bitter taste which I don’t like and if PrEP causes it that will be the end. (GD Males 13–17 years; South Africa)

Most participants said that if the pill was bitter/sour, they were unlikely to take it, irrespective of its effectiveness. Specifically, some participants made suggestions for pills that would be acceptable to them:

If PrEP is not bitter because something bitter is always problematic and PrEP tablets they bring are sweet and chewable. I can take it for even a year or three…bitter tablets are very problematic and not for me. (IDI Male 17 years; Uganda)

### Size of the Pill

Several participants expressed a fear of the discomfort of swallowing large PrEP tablets and thought they should break the tablets into two for easy swallowing. For some, the perception and the concern was that large tablets could cause choking and eventually kill someone.

The problem will arise if the tablets brought are big in that when you swallow it can choke you and you die. (GD Males 13–17 years; Uganda)

I even told the doctors that for me it’s hard to swallow the pill because I am not used to bigger pills, I am used to smaller pills. (IDI Female 22 years; South Africa)

### Side Effects of the Pills

Participants expressed a fear of the unknown consequences of taking PrEP, ranging from general body weakness to lowered sexual performance. These fears were based on information they had gained from discussion in their communities which fed their fears.

Feeling drowsy…Fatigue, and…Being lazy like doing nothing and always sleeping. Yeah. I am scared of things like that or maybe let’s say it gets to my testicles and does things that [unclear], you see? Cos I’m very careful towards my sperm. (GD Males 13–17 years; South Africa)

Other narratives revealed concerns about side effects related to skin irritation and ulcers leading to skin and stomach cancers.

There are some drugs you swallow and they bring “ebirogologo” [skin reactions/rash], so am concerned PrEP would also cause such side effects. (GD Females 13–24 years; Uganda)

(b) Interpersonal-level barriers

### Parental Influence

Participants expressed fear that their parents would find out that they were taking PrEP and therefore know they are sexually active. Sex at a young age (especially before marriage) is disapproved of in most sub-Saharan African cultures, and participants under the direct care of their parents revealed they would not take PrEP for fear of their parents discovering the pills.

The issue is on taking tablets every day and living with your parents. Where would I keep the tablets where they will not find them? If they come across them, it will be disaster at home. They will start saying let’s go to the hospital if you are sick. (GD Males 16–18 years; Zimbabwe).

I might accept to take PrEP but my parents might discourage me from taking it thinking I may engage in risky sex. Sometimes the women might discourage their husbands to take PrEP fearing they may go and have sex with other women yet the men are willing to use it. (GD Males 19–24 years; Uganda)

### Absence of a Sexual Partner or Faithfulness

The absence of a sexual partner and not being in long-term relationships were viewed by some participants as a barrier to PrEP uptake, as there would be no reason to take PrEP daily when there was no plan for sexual activity. Some of the participants who had partners and were choosing to be faithful to one sexual partner mentioned they would have no reason to take PrEP. These could potentially affect uptake and adherence to PrEP.

(c) Community-level barriers

### Myths and Misconceptions

Some participants confused PrEP with Post-Exposure Prophylaxis (PEP) and, in addition, did not understand how it could work to prevent infection. For example, there was a misconception about the dangers of taking medication when you are well.

When you take medicine when you are not sick, the medicine becomes poisonous in your life. Some may say if you are taking PrEP, you are taking poison which may bring you other illnesses. Therefore, you may find yourself leaving the medicine [PrEP]… (IDI Female 18 years; Uganda)

Furthermore, information from friends and peers was a key source of misconceptions yet they were their primary source of knowledge and support. When friends and peers disapproved of PrEP use, it was perceived as being unlikely to be used.

I think the youth of today, what would discourage them is that they value their friend’s opinion the most. So a person would tell a friend “I am taking such and such, pills [PrEP] and then the friend would say why are you taking this thing?” You see. So it is as if they are held by peer pressure. A person can’t do anything for him/herself, they do it to please others, before they do anything, they first seek the approval of their friends and if negative, they stop. (GD Males, 18–24 years; South Africa)

Parents were not favored as a source of knowledge because young people believed their parents would link PrEP to sexual initiation and early engagement in sexual activity. Some believed that some parents would perceive the introduction of PrEP to their children through the research project as a gateway to engage in risky sexual behavior.

### Misconceptions About PrEP Use

Participants associated PrEP with many “terms and conditions” required to take the drug. By taking PrEP, they anticipated that one should have enough money to buy everything their body demands, including ensuring that they eat well when taking the medication.

Like maybe I wake up in the morning then I am late, I must go to school, I don’t even eat food, obvious if you take, you must eat first isn’t it before taking pills. (IDI Male 16 years; South Africa)

The other thing I know, some people may not get the diet required to take the medication. At times you can be feeding poorly; eating “kikomando” [beans and chapattis] yet it seems it requires eating well [having a balanced diet]. (IDI Male 19 years; Uganda)

In the Ugandan context, “eating well” is equated to eating a variety of expensive foods like Matooke [banana], rice, chicken, meat, fish, milk, fruits and vegetables. In contrast, eating beans and chapattis (flour, water and oil pancakes) suggests consuming a less nutritious and inexpensive diet. Some participants mentioned they would not take PrEP because they were unable to meet dietary requirements. Young people mentioned other medicines like antimalarial prophylaxis and ARVs, that health workers recommend taking with specific foods like milk.

(d) Institutional-level barriers

### Medicine Stock-Out

Stock-out of medicine from the health facilities was reported as a potential barrier (this was mentioned most frequently in Uganda). Participants commented that health facilities lack essential medicines, and when people go to the health facilities, there is nothing available, and they are advised to buy from expensive private pharmacies. This discourages them from taking medicine.

What may make it difficult for one to use PrEP is when she goes to the hospital to get PrEP and they tell her that it’s not available or when they tell her to come back another day. (GD Females 20–24 Years; Uganda)

What if it [stock-out] happens for PrEP remember since I have been taking the daily…, let’s say I am taking my PrEP daily and then it happens that now I am out on pills, there are no pills [at the clinic or PrEP dissemination point]. This is going to ruin my cycle [of taking pills/adherence]. (GD Males 18–24 years; South Africa).

### Long Waiting Times at the Clinics/Busy Health Workers

Participants revealed that they preferred quick access to PrEP without having to spend long hours in queues waiting to receive PrEP. Instances when facilities are busy and they are required to wait for a long time was seen as a barrier to using PrEP.

The problem is the doctor may be attending to people slowly and yet there are many patients. I don’t want to sit there waiting to get a service so I will just give up and leave. Other people join in and the lines become long. (IDI Male 17 years; Uganda).

### Attitude and Behaviors of Health Workers

Some of the participants were concerned about the attitude of health workers when they are seeking services. This was viewed as a major barrier because they thought if the health workers are rude to the clients, they might not find it conducive to collect PrEP. Some, especially younger participants, feared that if those dispensing the medication are known to them, they might talk about them in the community to the extent of reporting them to their parents.

At times, the manner of approach and how they [health workers] explain things to you when they give you something, they are very judgmental. They don’t respectfully ask you questions nor do they make you understand. (GD Females 18–24 years; South Africa).

(e) Structural-level barriers

### Cost of PrEP

Most participants were concerned that if PrEP was not free they will not manage to buy it because most participants had an unstable source of income:

I think there are two reasons that would make my friends fail to take PrEP. The first reason is, if it is to be priced, he may fail to buy it from the shop. (GD Males 13–17 years; Uganda)

People would want to take the pills, but the cost of the pills may be a barrier. Some pharmacies want US Dollars for the pill and one may not afford it, that’s another challenge. (GD Females 19–21 years, Zimbabwe).

### Mode of Administration

Participants shared their fear of taking tablets daily and would prefer an injection, implants or syrups. As oral PrEP would be the most accessible and easy to administer, participants acknowledged its limitations and expressed a preference for long-acting PrEP options such as injections and implants.

For me on demand is tricky because at times you might be with your boyfriend and you left the pills at home and you want to have sex right there and then and you weren’t taking PrEP every day and it becomes difficult. So, the best option would be getting an injection in advance like for 6 months, that will be better. (GD Females 19–21 years; Zimbabwe).

Some, however, did not want to take anything:

Even if you just bring tablets and put them there, I just vomit. Some may fear to take PrEP tablets and say that “I rather fall sick with the thing [HIV] than taking those tablets.” (GD Females 13–17 years; Uganda)

### Accessibility Concerns

Participants noted that if the access point for PrEP is not within easy reach, they might find it a problem to take PrEP. This was viewed in terms of points being far away from the users and requiring transport and time.

What may make it difficult for me to take PrEP, in case they decide to put PrEP in [the hospital] and we all have to get PrEP from there and when a person wants to go there it can cost around ten thousand shillings which I don’t have, then I wouldn’t go. (GD Females 20–24 years, Uganda)

### Facilitators to PrEP Uptake Among AYP

1. Individual-level facilitators

From the IDIs and GDs, we identified two individual-level facilitators: (a) high HIV risk perception and (b) preventing HIV acquisition and desire to remain HIV negative.

### High HIV Risk Perception

Some of the participants mentioned that perceiving themselves as being at risk of contracting HIV was a key facilitator to PrEP uptake. They mentioned that aspects like not knowing the HIV status of one’s partner, dislike and fear of going for an HIV test by their partners, created a fear of becoming infected. The introduction of PrEP was therefore viewed as an opportunity to reassure someone that they are protected and will not be infected.

Haa PrEP will make me feel comfortable because whenever I have sex I begin to worry whether my partner has transmitted to me the virus which causes a lot of stress. With PrEP I will be certainly sure that all is well. (IDI Male 19 years; Zimbabwe)

Distrust of a partner and the increased chance of acquiring HIV were reported as a motivation to initiate PrEP.

I would use it [PrEP] because I no longer trusted the father of my child before I fell pregnant. He was boring and disgusted me. His phone was always busy and I do not know what he is doing with other women. I had that mentality that he may be sick and I do not know what he is doing and when. (GD Females 18–24 years; South Africa).

### Preventing HIV Acquisition and Desire to Remain HIV Negative

Some participants mentioned that the desire to remain HIV negative was a potential facilitator to consider PrEP use. These participants expressed a willingness to take PrEP due to the nature of sexual activities they engaged in or that existed in their communities. Most of them viewed PrEP as a breakthrough strategy to prevent acquisition of HIV.

I will swallow PrEP because I know that I will not contract HIV. I may meet a girl who has every sexually transmitted disease including HIV but PrEP will help me not to get HIV after I have had sex because for its case it does not cure. For other STIs, you can get medication and recover but not HIV. (GD Males 13–17 years; Uganda)

Ehhh, honestly guys, to tell the truth, for now this [PrEP] is the best thing that we have to stay negative and prevent this disease [HIV]. I am sitting here and talking about the way I am going to protect myself from HIV and that’s for me is like the icing on the cake, the cherry on top, seriously. (GD Males 18–24 years; South Africa)

PrEP was seen as a safer prevention strategy that would build intimacy for those who had difficulty to utilize other HIV prevention like condoms. Some participants had concerns and reservations for using condoms such as a decrease in sexual pleasure and dislike of condoms by sexual partners and viewed PrEP as a possible prevention alternative.

There is a time when my boyfriend drinks a lot. So when I go to see him, I know that we will sleep together [have sex]. So I believe I will need PrEP, because he does not hear my voice when I say let’s use a condom. He asks “why now, why do you want a condom?” (IDI Female 20 years; South Africa)

Use of condoms will definitely reduce since most people use condoms to prevent HIV. So if I get PrEP that I can use to prevent HIV infection without using a condom, then condom use will be unnecessary. (IDI Male 18 years; Zimbabwe)

(2) Interpersonal-level facilitators

### Peer Influence

Most participants expressed willingness to take PrEP if other people especially their peers were taking PrEP. Participants acknowledged that they would be inclined to take PrEP if they realized others took the pills and nothing happened to them after taking PrEP.

The best way for them [peers] to use it is when they see me using it…Yaahh, they want protection, they need to see me having used it and am alive. Then they will say let’s go boys and we get them [PrEP pills] together. (GD Males 19–21 years; Zimbabwe)

### Social Support and Care for PrEP Uptake

Support, care and encouragement mainly from family, supportive partners and friends were also viewed as an important facilitator for PrEP uptake. The participants remarked that receiving care and support (financial or material) from immediate family would encourage them to use PrEP.

I think that it’s the circle of friends that we keep that should encourage you to take PrEP because the people that should care and support you first are your family and then your friends because if they don’t support you they will think that you are sick and dying and want to kill them as well. (GD 18- Females 24 years; South Africa).

(3) Community-level facilitators

### Adequate PrEP Information and Sensitization

Some participants mentioned that receiving adequate PrEP information and sensitization was a prerequisite for taking PrEP. Participants shared that information about PrEP needs to be disseminated in simple language that people can easily understand.

What will make it easy is being well informed so that we will have in-depth knowledge that this medication can prevent us from HIV infection. (GD Males 13–15 years; Zimbabwe).

Some participants mentioned that when information about PrEP is made available, with opportunities to discuss PrEP, making it known in the community, then the likelihood of PrEP uptake would increase.

### Evidence of PrEP Efficacy and Safety

In addition to adequate PrEP information, the participants noted that receiving information and evidence that PrEP works and is highly effective could facilitate PrEP uptake.

If they notice that people are not dying or if they go to people who have used PrEP and they are not affected by PrEP or not infected with HIV, young people will take it. (IDI Male 23 years; Uganda)

Since many participants were PrEP naïve, they had a perception that PrEP was still under experimentation and research. They considered that obtaining evidence to show that PrEP was safe and effective would encourage PrEP uptake. Participants narrated that if they are given examples of PrEP working well, many more people would be motivated to take it.

(4) Institutional-level facilitators

### Convenient and Responsive Services

At the institutional level, as in many situations, health care services are often at a significant distance from the people, some participants mentioned that access to PrEP needed to be designed in an adaptive and innovative way, so that many young people who would benefit from it and receive PrEP.

If its near let’s say, a government hospital or nearby health centers, I would move and go looking for PrEP. It will be easy for me when I am getting it [PrEP] from near…But if it is in [mentions a distant place], I will have to “put in” [incur a cost] transport to go get it yet at times we don’t have money. I don’t work money is hard to get. (IDI Female 17 years; Uganda)

Provision of PrEP with convenient local access, creation of different platforms and PrEP service providers such as community health workers who are responsive to provide health services to adolescents was a considered a potential facilitator of PrEP uptake.

### Provision of Appropriate and Sufficiently Resourced Services

In relation to convenient and responsive services, participants noted that a facilitator to PrEP uptake was for it to be delivered in an appropriate and sufficiently resourced manner to minimize stock-out challenges, and including provision of information and sensitization through promotions and campaigns to support demand creation.

It may be good for dispensing in the clinics so it is easy to obtain. If I go to this clinic when it [PrEP] is finished, then I can go to another clinic and get it. (IDI Female 21 years; Uganda).

…I think it requires campaigns to be done in the population like you moving around encouraging young people to take PrEP and you people telling us why it’s good. (IDI Female 21 years; Zimbabwe).

(5) Structural-level facilitators

### Access and Availability

Participants reported that having PrEP available in places closer to the young people would be a great advantage and a facilitator to PrEP uptake.

Let us say that they have initiated PrEP at Entebbe hospital it will be hard to get it but if you distribute it in all the communities one can easily get it…It will be easy for me to access it if it is in the nearby health centers, and I can pick it at any time because sometimes I may not be able to walk long distances when I am too busy. (GD Males 19–24 years; Uganda).

Linked to provision of appropriate services, accessibility and availability were important attributes of PrEP uptake and some participants noted that PrEP would be easily used if they were comfortable with the way it is delivered as a youth-friendly service, convenient to obtain and flexible to access.

At least if they manage to use shopping centers which are easily accessible for many because no one can say I can’t go to the shopping center. Many people always go to the shops, in hospitals or near these hospitals people can go there. (IDI Male 21 years; Zimbabwe)

### Costing of PrEP

Structural facilitators included providing PrEP free of charge. For some adolescents and young people in these communities, providing PrEP as a free service would be a big facilitator for PrEP uptake. To these participants, anything that comes with a cost is quickly shunned.

I think that they will be happy because no person likes to die of AIDS. If they see that the pills are available for free they will be happy because some may not get money to buy Protector [condom brand] and end up having unprotected sex. (GD Females 13–15 years; Zimbabwe).

For many AYP, who are often financially vulnerable, receiving PrEP at no cost would facilitate its uptake.

Some hospitals can be selling yet others are not selling and the one next to you is selling. If you know that you are not sick you will say let me go do something else with that money and you overlook your health. Personally, if PrEP came and it is not for paying then it will be very easy for me. (IDI Female 17 years; Uganda).

## Discussion

PrEP as a biomedical HIV prevention strategy can minimize HIV acquisition among AYP in sub-Saharan Africa. Understanding the barriers and facilitators to PrEP uptake is critical to enhance the successful utility of PrEP among AYP. We used the social ecological model to explore key barriers and facilitators to PrEP uptake among mostly PrEP naïve AYP aged 13–24 years in sub-Saharan African settings.

The SEM allowed us to reflect on the interdependent nature of our findings at multiple levels and interrelationships (McLeroy et al., 1988). Some of the findings were overlapping at the different levels of the model. The way AYP responded to PrEP as a new biomedical HIV prevention strategy was embedded in different structures ranging from individual through to structural factors. The findings highlight important implications for addressing the barriers and enhancing the facilitators to PrEP uptake among AYP at the different levels of the SEM.

At the individual level, we found new perspectives on barriers to PrEP uptake compared to the existing literature, such as the complexities of timing of taking PrEP and PrEP characteristics. People may be willing to use PrEP, but due to their busy schedules may find it difficult to adhere to taking it as prescribed, which thus hinders its effective use. For example, the fishermen of Lake Victoria may not take PrEP because of their mobile lifestyle (Mack et al., 2014). Keeping to a regular time for taking PrEP was also a concern for the school going children in our study. Our findings further confirm that stigma, expected side effects, and pill burden were barriers to PrEP uptake, consistent with findings from other studies (Holloway et al., 2017; Patel et al., 2016). These are important issues to consider for successful PrEP implementation and utilization in resource constrained settings where treatment for prevention is not a common practice.

Participants’ perception of HIV risk was a facilitator of PrEP uptake and their preference was for on-demand PrEP especially when they felt at low risk of HIV and did not want to commit to a daily PrEP regimen. This finding is similar to those found in other previous studies among MSM (Lorente et al., 2012; Molina et al., 2017). On-demand PrEP would address their concerns over daily use and possible adherence challenges in the context of not having routine schedules.

Our study unpacks other PrEP characteristics and properties including the cost of PrEP as a potential barrier to uptake. Additionally, the size, taste and smell of PrEP pills should be considered alongside the favorable properties of PrEP agents (safety, tolerability and long-lasting activity with convenient dosing) in developing the next generation PrEP to make PrEP attractive and convenient to use by the intended end users (Abraham & Gulick, 2012).

The findings highlight important implications for PrEP uptake among AYP at the interpersonal level. Most participants were under the care of their parents/caregivers or were in a relationship, therefore could not easily make independent decisions and also had a lack of privacy. Even though most would have been willing to take PrEP, they felt restricted, including the fear of being discovered as sexually active by their parents. This suggests that systemic introduction of PrEP to the community and sensitization of key stakeholders (parents, peer leaders and other influential people in the community) to solicit for their advocacy and promotion of PrEP for HIV prevention is important (Shah et al., 2019). This would thus enable adolescents and young people to embrace the use of PrEP as an HIV prevention measure.

Our results also revealed peer influence as an important facilitator of PrEP uptake, alongside social support. Participants noted that PrEP was coming in at a time when many AYP felt that other biomedical HIV prevention strategies were not suitable for the adolescent population. It is imperative to note that decreased HIV risk perception has been associated with poor adherence and low interest in prevention interventions such as PrEP or microbicides and has the potential to increase risky sexual behaviors (Pettifor et al., 2013).

Our findings showed existing misconceptions about PrEP at the community level, which were perceived as a potential obstacle to uptake. Previous studies among transgender adolescents and at-risk populations in Peru and Kenya found similar results (Fisher, Fried, Desmond, Macapagal, & Mustanski, 2017; Galea et al., 2011; Mack et al., 2014). Many AYP may be discouraged by the community reflections on the prospect of potential medication side effects and the skepticism of PrEP use in relation to its efficacy to prevent infection as reflected in a previous study in France and Canada among MSM (Molina et al., 2017). We recommend providing adequate information and sensitization for communities, including AYP, on PrEP efficacy and safety that is still limited in this setting but will importantly facilitate informed decision making on PrEP uptake. Our research group is currently conducting a clinical trial to evaluate the efficacy and safety of different PrEP drugs (oral Emtricitabine/ Tenofovir Disoproxil Fumarate and oral Emtricitabine/Tenofovir Alafenamide), including timing of doses, among young people 13–24 years in Uganda and South Africa. Community-based approaches to PrEP sensitization and roll-out, such as community education campaigns, may be an important step toward reducing PrEP barriers and achieving increased PrEP acceptance levels by enhancing the facilitators (Golub et al., 2017).

At the institutional level, the lack of training in PrEP provision and negative attitudes toward PrEP can influence health worker perspectives, prejudicial beliefs and PrEP provision to AYP. Therefore, the utilization of non-prescribing providers can help overcome the institutional barriers to PrEP uptake (Pinto, Berringer, Melendez, & Mmeje, 2018).

It should be noted that AYP expressed willingness to take PrEP once provided at a service that is low cost, regularly available and easily accessible, suggesting that PrEP provision should be tailored for AYP at the structural level (Yakob & Ncama, 2016). Other studies have shown that cost and access are major barriers for PrEP uptake among other populations (Pérez-Figueroa, Kapadia, Barton, Eddy, & Halkitis, 2015; Young et al., 2014). Making pill access and pickup points more reachable will make PrEP more widely available and can help to mitigate concerns of failing to reach for the pills when needed.

Overall, the majority of participants welcomed the concept of PrEP and were curious to know when it will be rolled out. PrEP as a breakthrough HIV prevention strategy for the AYP relates to the empowerment, autonomy and discreetness PrEP provides to adolescents in relation to HIV prevention. This is critical in sub-Saharan Africa settings where intergenerational sex among AYP is common and associated with reduced ability to negotiate safe sex, low condom use, unsafe sexual behavior that increases the risk of HIV infection (Luke, 2003). The high HIV prevalence rates among AYP in sub-Saharan Africa highlight the urgent need for effective and targeted PrEP strategies (Baeten et al., 2012; Shisana et al., 2014). Therefore, addressing the identified barriers of PrEP uptake in this underserved population is critical (Bekker & Hosek, 2015).

These study findings offer new perspectives into the likely barriers and facilitators to PrEP uptake among PrEP naïve adolescents and young people in sub-Saharan Africa and future studies should examine how the barriers and facilitators at the different levels of the SEM affect adherence when PrEP is rolled out among AYP (Pinto et al., 2018).

Study limitations include the study being conducted in PrEP naïve communities where the concept of PrEP was confused with other constructs like school study/reading time, and post-exposure prophylaxis. As the study participants were PrEP naïve, we identified perceptions rather than actual behavior in a non-treatment study population. Similarly, despite our large sample we did not carry out comparative analyses by age, gender and country which are crucial areas for future research. However, our findings provide important insights into potential barriers and facilitators of PrEP uptake among AYP in low-resource settings. Future studies in our context need to evaluate the potential barriers and facilitators to PrEP uptake from service providers that provide adolescent-friendly services, healthcare workers and policy makers perspectives.

In summary, there is a range of barriers and facilitators that would affect PrEP uptake. When addressed, PrEP will serve as an additional HIV prevention strategy for AYP who are potential PrEP users (Bekker & Hosek, 2015). The development of appropriate youth-friendly services and programs providing free or inexpensive quick access using various platforms and providers with minimal PrEP stock-out, friendly health worker contact, privacy and confidentiality will support PrEP uptake and utilization. Additionally, providing information and mitigating the barriers at all levels will encourage PrEP uptake and eventual adherence. Future multi-level interventions should consider the social and structural drivers when designed and focus on ways that can inspire PrEP uptake and limit the barriers.

## Electronic supplementary material

Below is the link to the electronic supplementary material.

Supplementary material 1 (DOC 61 kb)
