# Supplementary material for: Exploring Perceived Barriers and Facilitators of PrEP Uptake among Young People in Uganda, Zimbabwe, and South Africa
Source: Arch Sex Behav. 2021 May 5;50(4):1729–42. doi: 10.1007/s10508-020-01880-y (PMC8213546; doi:10.1007/s10508-020-01880-y)
Supplement: Supplementary file 1 — Supplementary material 1 (DOC 61 kb) [file 10508_2020_1880_MOESM1_ESM.doc]

**Appendix A**

| **CHAPS Qualitative Guide** |
| --- |
| **Thank you for agreeing to be part of this study In the next hour we will be talking about your perceptions on HIV risk and sexual risk behaviour among young people. We will also discuss what you know about Pre-exposure prophylaxis (PrEP) and what you think will motivate or hinder young people to use it. Please feel free to ask me for any clarity regarding what we will be discussing and remember that there is no wrong or right answer.** |
| **Background information**   - Please tell me a little bit about yourself? (hobbies, friends, where you hangout for fun) - Tell me about the last time you tested for HIV. Why did you decide to test for HIV? Where did you test for HIV? What was your experience of testing for HIV? If NEVER tested for HIV. What are the reasons you have never tested for HIV? |
| **HIV RISK PERCEPTIONS**   - How do you think young people that you know are at risk of contracting HIV? Probe: which group of people do you think are more at risk: sex, gender, age?​ - How do you think you are/ are not at risk of contracting HIV? - Please tell me about instances where you thought that you are at more risk than others?​ - Please tell me about ways that young people (including yourself) are using to protect against HIV. |
| **PrEP in general**   - What have you heard about Pre-exposure prophylaxis (PrEP)? (antiretroviral medication used for HIV prevention) - Probe for who told them and what they heard about it. - Please tell me about what you know about PrEP or about how it works. - How/where did you first learn about PrEP? - Tell me what you think people in your community feel about taking a medication every day to prevent HIV? - How would you feel about taking a medication every day to prevent HIV infection? - How would you feel about taking a medication only on-demand to prevent HIV infection? |
| **Acceptability of PrEP**   - What would be the reasons your close friends would want to take PrEP? - What would be the reasons you would want to take PrEP? - What would be the reasons you would NOT want to take PrEP? What would be the reasons your close friends would NOT want to take PrEP? - Please tell me about the instances where you would have needed on-demand PrEP/ it would have been beneficial for you to take it? - What do you think will make it easy for you to use PrEP daily? - What do you think will make it difficult for you to use PrEP daily? - Tell me what concerns or fears you may have about using PrEP? |
| **Social and community concerns**   - What are possible social concerns that may discourage you from taking PrEP? (probe about close friends) - Tell me about the stories/rumours/gossip about PrEP in your community? (probe amongst your friends and family members) - What stories have you heard about girls/boys who use PrEP or participated in PrEP studies? - What influence do you think these stories will have on your decision to use/not use PrEP or to participate in PrEP studies? |
| **Behavioral change after PrEP**   - How would having PrEP available change your sexual behaviors? - How would having PrEP available change your friends’ sexual behaviors? - How would taking PrEP change your use of condoms? - How would taking PrEP change your friends’ use of condoms? - What are things we might be able to tell your friends to prevent them from increasing their risk behaviors if PrEP were available? What might we say? |
|  |
| **Health Care Provider concerns**   - What are possible concerns about health care providers that may discourage you or your close friends from taking PrEP? - How can these challenges be eliminated? |
| **PrEP Characteristics**  ***Side effects***   - What do you think taking PrEP when you are HIV negative will do inside your body? - How do you think using PrEP may change anything in your life or in your daily life? - How do you think taking PrEP will affect your sexual experience?   ***Duration of taking PrEP (*Days, weeks, years, lifetime)**   - What length of time do you think is reasonable for one to take PrEP?   ***Effectiveness***   - What percentage of effectiveness should PrEP have in order for people to use it? - How effective do you think PrEP is in preventing HIV infections?   ***Frequency of administration (once a day vs. before sex acts)***   - Why would you prefer taking PrEP daily? - Why would you prefer taking PrEP on-demand (before sex acts)? - How many pills do you think are appropriate to take daily? Why do you think so?   ***Cost***   - How willing will you be to use PrEP if you were to pay for it? How much would you be willing to pay for PrEP? - How willing you will be to use PrEP if it was available for free?   ***Places of dissemination (pharmacy, clinic, adolescent/youth centre, doctor’s office)***   - Tell me about where you think you can get PrEP when you need to use it? - How easy do you think will be for you to get PrEP? Tell me why you think so. - Where would you prefer to access/get PrEP?   ***Person who dispenses (doctors, nurses, peer counsellor, HIV counsellor)***   - Who would you prefer to dispense PrEP for you and why? |
|  |
| **Partner Dynamics**   - Tell me how many partners you have? Tell me about your partner/s (age, gender, sexual orientation) - How do you think using PrEP may change your relationship/s in any way? - How do you think your partner/s will react about you taking PrEP or your participation in PrEP studies? - What do you think will make it easy to reveal/conceal to your partner/s your use of PrEP or participation in PrEP studies? |
